# Cholesterol Embolization Syndrome From Penetrating Aortic Ulcer

**DOI:** 10.7759/cureus.8670

**Published:** 2020-06-17

**Authors:** Jennifer Nickol, Theodore Richards, Jared Mullins

**Affiliations:** 1 Internal Medicine, Magnolia Regional Health Center, Corinth, USA; 2 Cardiology, Magnolia Regional Health Center, Corinth, USA

**Keywords:** penetrating aortic ulcer, cholesterol embolism syndrome, blue toe syndrome, cholesterol crystals, acute aortic syndromes

## Abstract

Penetrating aortic ulcer (PAU) is an important, albeit, rarer cause of embolization to internal organs and distal extremities. Embolization occurs as a result of the disruption of cholesterol deposition in the wall of the aorta by a PAU. The classic presentation of cholesterol embolization syndrome (CES) includes pain, pallor, poikilothermia, paresthesia, and paralysis with intact pulses. The patient will classically have livedo reticularis or “blue toes.” We present a case of a patient who presented to the emergency department with the complaint of a painful, blue toe. The patient had intact distal pulses on exam with the distal 2/3 of the first toe having a markedly blue/black color with livedo reticularis spreading proximally on the other 1/3 of the toe. CT angiogram with runoff to the lower extremities revealed a 3.6-cm infrarenal abdominal aortic aneurysm with a 5-mm penetrating aortic ulcer with a three-vessel runoff to the distal lower extremities. The diagnosis of CES secondary to a PAU was made. While thrombotic embolization from PAU causing acute limb ischemia is less common, it is well described. In contrast, cholesterol embolization from PAU remains a rare phenomenon without adequate treatment options.

## Introduction

Penetrating aortic ulcer (PAU) is an important, albeit, rarer cause of embolization to internal organs and distal extremities [1-3]. This embolization is through a mechanism of cholesterol embolization that can arise from many atherosclerotic disease processes, including PAU, and is known as cholesterol embolization syndrome (CES) [4]. These emboli can often be confused for cardioembolic or thrombogenic sources if PAU and CES are not carefully considered within the differential. Important clinical signs that point towards microembolism include the classic signs of arterial embolization: pallor, pain, poikilothermia, paresthesia, and paralysis. In CES, however, one will find intact pulses and possibly livedo reticularis [4]. We present a case of a penetrating aortic ulcer causing CES.

## Case presentation

A 72-year-old white male with a past medical history significant for emphysema, tobacco abuse, Alzheimer’s dementia, coronary artery disease, hypertension, hyperlipidemia, cryptogenic cerebrovascular accident (taking warfarin), and permanent pacemaker implantation due to sinus pauses was admitted to the hospital for a painful, blue toe. He presented to the emergency department with the chief complaint of a sore toe for one week that was beginning to turn black. He had not experienced any fever or chills, chest pain, palpitations, shortness of breath, or purulent material being expressed from the toe wound. No history of trauma to the toe was elicited. Physical exam was remarkable only for a 1 cm x 1 cm black eschar at the tip of his left great toe with a surrounding blue hue and no purulence. The toe was cold and painful on palpation. Active and passive range of motion was intact. Dorsalis pedis and posterior tibialis pulses were 3+ bilaterally.

**Figure 1:**
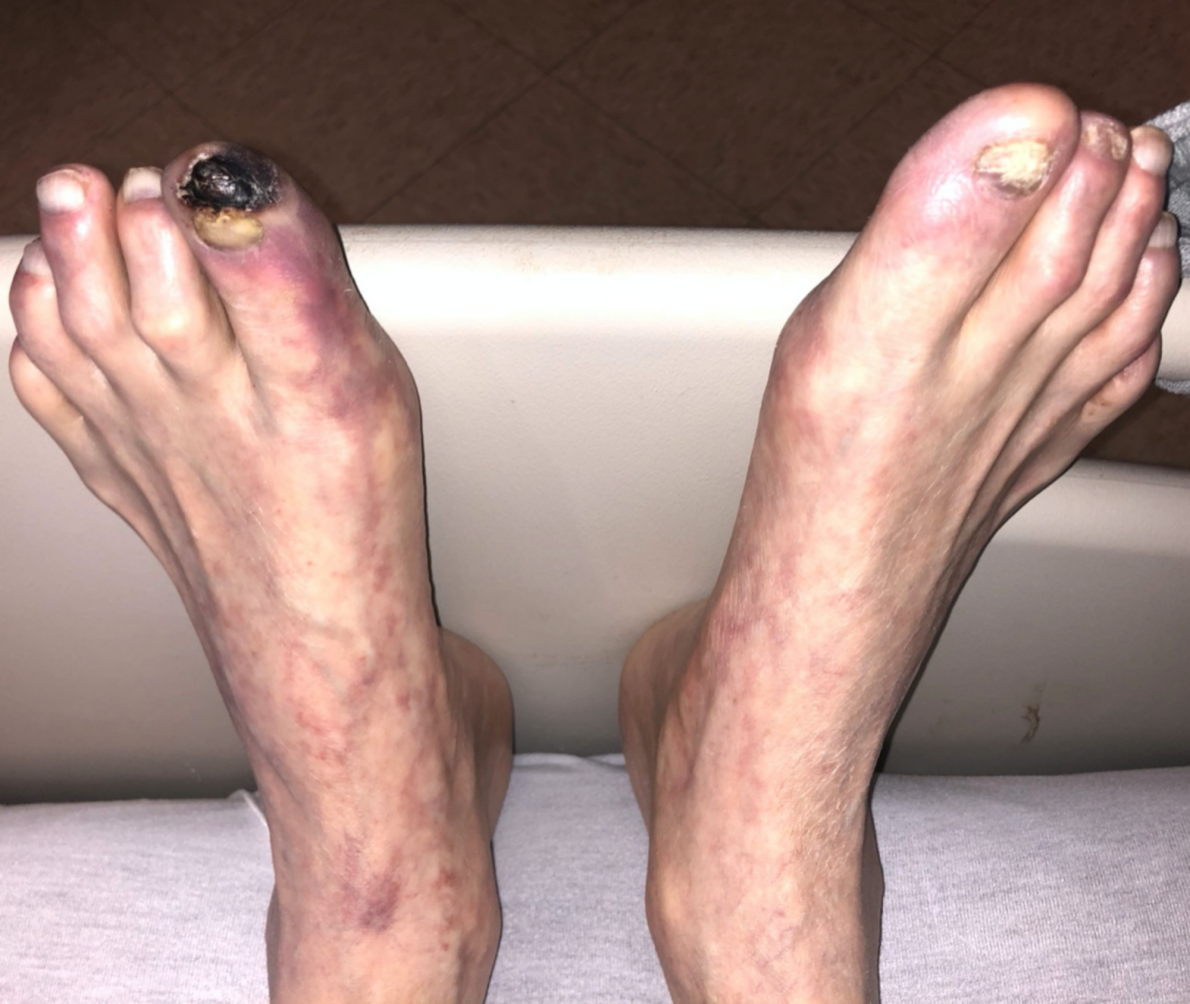
"Blue" left toe with necrotic eschar and surrounding livedo reticularis.

The initial laboratory workup was unremarkable except for a supratherapeutici nternational normalized ratio (INR), and warfarin was held. CT of the foot was performed that revealed an obvious defect on the distal first phalanx consistent with the eschar seen on exam, but was negative for osteomyelitis or signs of cellulitis. He was started on cephalexin for anti-microbial coverage. On hospital day 2, the toe necrosis appeared to have spread, and there was now a livedo reticularis appearance to the toe (Figure 1). At this time, infection was felt unlikely and cephalexin was discontinued. Blood cultures showed growth of Staphylococcus epidermidis, but due to lack of systemic symptoms these were considered contaminant. Repeat blood cultures remained negative without further antibiotics. At this juncture, an arterial or embolic source was highly suspected, and lower extremity arterial Doppler studies and transthoracic echocardiogram (TTE) were obtained. The lower extremity Doppler studies were normal bilaterally with triphasic blood flow. The TTE showed a normal left ventricular ejection fraction, trace mitral and tricuspid regurgitation, and a dilation of the ascending aorta measuring 4.4 cm. There was no evidence of endocarditis or embolic source in this study. Despite the negative arterial studies, there was still a high suspicion for an arterial or embolic source.

**Figure 2:**
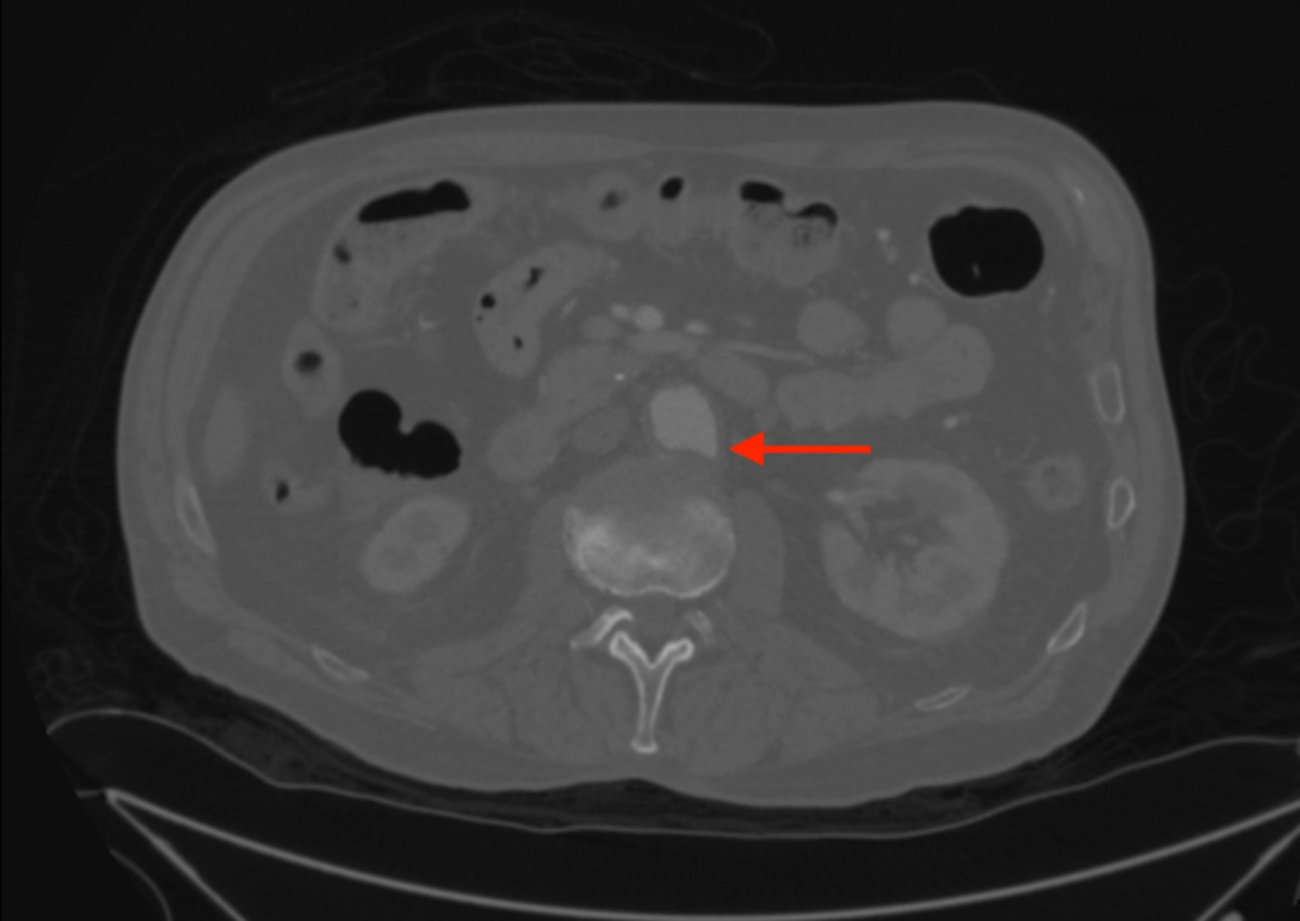
CT reveals a 5-mm penetrating aortic ulcer and 3.6-mm aneurysm of the infrarenal abdominal aorta (arrow).

A pacemaker interrogation revealed that he had been in normal sinus rhythm since the pacemaker was inserted one year prior. A CT of the aorta with runoff was performed (Figure 2). This revealed a 3.6-cm infrarenal abdominal aortic aneurysm with a 5-mm penetrating aortic ulcer and three-vessel runoff to both lower extremities. He was evaluated by cardiothoracic surgery for possible surgical intervention of PAU. However, he was considered to be a poor surgical candidate for open or endovascular repair of the PAU, nor did he meet the criteria for repair of the abdominal aortic aneurysm. 

He was previously taking 80 mg of atorvastatin prior to arrival at our facility, and this drug was continued. We continued previous dose of 2.5 mg of lisinopril and started 25 mg of metoprolol succinate for prevention of progression of the abdominal aortic aneurysm and PAU. Due to the severity of this patient’s atherosclerosis and his prior stroke, we chose to start him on 81 mg of aspirin and 75 mg of clopidogrel for further atherosclerotic cardiovascular disease (ASCVD) risk reduction. Warfarin was stopped due to the potential to provoke cholesterol embolization. Ultimately medical therapy was unsuccessful, and the patient required amputation of the left leg. 

## Discussion

PAU is the ulceration of an aortic atherosclerotic plaque penetrating the internal elastic lamina into the aortic media and accounts for 2%-7% of all acute aortic syndromes [5,6]. The natural course of PAU and its treatment remains controversial and unresolved [2,3,5-8]. The complications of PAU include pain, development of intramural hematoma, aneurysm formation, and progression to rupture or dissection [5,6]. While embolization from thrombus formation with PAU is described in the literature, cholesterol embolization is rare [1,2]. There have been cases describing distal embolization via thrombus formation, most often as result of abdominal PAU, but we identified only one other case of atherosclerotic embolization and blue toe syndrome related to infrarenal PAUs [1,2,9]. The mechanism of PAU cholesterol embolization is due to cholesterol plaque rupture, distal embolization of plaque debris (cholesterol crystals, platelets, and fibrin), occlusion of small arteries, inflammation response to cholesterol emboli, and ultimately necrosis [4].

Treatment options must take into account both CES and PAU. Unfortunately, neither PAU nor CES has guideline consensus on treatment [2-9]. With respect to the treatment of PAU, the embolic source of the cholesterol crystals, there is no standard size or approach to surgical intervention in asymptomatic patients. Treatment is recommended in patients with aneurysm expansion, rupture, embolic symptoms, or uncontrolled pain [8]. Thoracic endovascular repair (TEVAR) is a well-described treatment option for PAU, but concerns exist given the atherosclerotic nature of the disease with respect to difficulty in obtaining access to the femoral artery [2,3,5,7,8]. Many authors describe the safety of TEVAR, in comparison to open repair, in pre-emptive treatment to prevent catastrophic event of dissection or rupture, while others advocate watchful waiting due to likelihood of benign course [2,3,5,6,8]. A recent retrospective analysis by Gabel et al. showed no predictive patterns for disease progression but suggested that early referral to vascular surgeon was associated with improved survival and decreased likelihood of progression [10]. In the case of CES caused by PAU, this phenomenon falls under embolic symptoms and therefore warrants surgical intervention.

Treatment of CES focuses on supportive care for the end-organ dysfunction caused by the cholesterol emboli and prevention of further emboli. Statin therapy is generally encouraged as a treatment strategy and has some weak evidence to support its prevention of further embolization [4,9]. Anti-platelet medications have no clear evidence for treatment or prevention,;however given the high risk of concurrent coronary artery and cerebrovascular disease it is reasonable to prescribe [4]. Although there has been no convincing evidence that anticoagulants have a causal relationship with cholesterol embolization, they are generally avoided. Due to the inflammatory pathogenesis of CES, strategies aimed towards this pathway may lead to future development in treatment progression. There have been successful case reports using cyclophosphamide, corticosteroids, and colchicine; however, no randomized control trials exist [4,9]. 

## Conclusions

Thrombus embolization caused by abdominal PAU is a cause of acute limb ischemia and may be treated using TEVAR. PAU causing CES, however, is a rare and difficult to treat entity. Morbidity is high in patients with CES and many undergo amputation. There are potential treatments with anti-inflammatory drugs on the horizon but guidelines are lacking. TEVAR can help prevent further cholesterol embolization and prevent catastrophic sequelae such as aortic dissection or rupture. Given the rarity of PAU causing CES and the lack of treatment guidelines for treatment of abdominal PAUs in general, we conclude that TEVAR would be a reasonable treatment strategy.
